# Supplementary material for: A multi- and mixed-method adaptation study of a patient-centered perioperative mental health intervention bundle
Source: BMC Health Serv Res. 2023 Oct 27;23:1175. doi: 10.1186/s12913-023-10186-3 (PMC10612159; doi:10.1186/s12913-023-10186-3)
Supplement: Supplementary file 1 — Supplementary Material 1 [file 12913_2023_10186_MOESM1_ESM.docx]

**APPENDICES**

**S1. Interview Guide**

**Semi-Structured Interview Guide: PATIENTS**

**Medication Optimization**

1. How was your experience with medication optimization?
2. Before beginning the intervention, did you know what medications you were taking and for what?
3. Do you frequently review your medications with your physician (PCP or geriatrician or psychiatrist)? How often do you have these medications reviews?
4. How effective was the medication optimization by our wellness partners in improving your mental health and emotional well-being?
5. How does medication optimization differ from what you have done in the past?
6. What were the advantages of medication optimization?
   1. What aspects of the intervention would make you recommend the intervention to someone else?
7. Did our perioperative wellness partner update you on medication changes?
   1. If so, what was communicated (e.g., rationale for medication changes) with you?
   2. What specific medications were optimized?
   3. When and how?
   4. Was it helpful?
   5. Was the medication changes effective in improving your mood/ anxiety and depression?
   6. Did you experience any side effects with particular meds that were optimized?
   7. What were the side effects? Was it communicated with the partners and updated accordingly?
   8. Did you share the optimized medication list with your provider? Any feedback from the communication? Were they aware and okay with the changes?
   9. Did you adhere to the new medication prescriptions as suggested by our partners?
      1. If so, how?
      2. If not, what problems did you experience?
8. Did you experience any other problems when the medication changes were made?
   1. If so, can you describe them further?
   2. Also, how could we address these in the future?
9. Do you have any other comments or suggestions regarding medication optimization sessions?
   1. What would have helped you the most during these medication optimization sessions with the partners?

**Behavioral Activation**

1. How was your experience with behavioral activation (values, goals, activity scheduling, activity tracking)?
   1. How did you feel about each of these aspects of behavioral activation?
   2. Did it take into account “what mattered most” to you? (for e.g., outcome/goals/care preferences with regards to Quality of life/functioning, enjoying life, connecting)
2. From your perspective, how effective was behavioral activation in improving your well-being?
   1. How effective was behavioral activation in easing your anxiety/depression surrounding surgery?
   2. Where did BA help the most: pre-, post-operative periods?
   3. What did you like most about BA?
3. Do you feel that the timing and frequency of sessions were appropriate?
   1. How many sessions did you have? (before and after surgery)?
   2. Did the sessions before surgery help you prepare for the surgery and ease your anxiety and stress around surgery?
   3. Did the sessions after surgery help your mental and physical recovery and well-being?
   4. Would you change the number of sessions or how often you had them (frequency)?
4. What are your thoughts on the format of the sessions?
   1. Do you prefer individual sessions or group sessions?
   2. Was telephone effective or do you prefer in-person sessions? Would zoom option be something you would have been comfortable with?
5. Was goal-setting/value formulation/activity hierarchy helpful to you?
   1. How did the goal <example> help you cope with anxiety and depression?
6. Would you be willing to use behavioral activation if you were to need surgery again in the future?
   1. If so, why?
   2. If not, why not?
7. What were the advantages of behavioral activation?
   1. What aspects of behavioral activation would make you recommend these practices to friends/family?
8. What were the disadvantages of behavioral activation?
   1. What made behavioral activation difficult to follow or adhere to?
   2. What did you like during these sessions? Please elaborate upon it.
   3. What did you dislike during these sessions? Please elaborate.
   4. What were your concerns and how can we improve upon addressing your concerns?
   5. Did you feel overwhelmed with behavioral activation at any point?
   6. What are some suggestions you may have to improve the acceptability and feasibility of BA?
9. How was your experience with the perioperative wellness partner delivering the BA intervention? Please elaborate on each of the below.
   1. During your interactions with the partner, did you feel that you were cared for and supported?
   2. Was the communication by your partner sensitive to your needs?
   3. Did you feel that the partner spoke with kindness?
   4. Did you feel the partner listened to you, your needs and acted upon accordingly?
   5. Did the partner express empathy in their interactions?
   6. Did you have a good relationship with your partner?
   7. Did you feel the partner was attentive?
   8. Did you feel the partner was responsive to you?
10. Did you have any concerns/comments about your partner that need to be addressed?
11. Do you have any other comments or suggestions regarding behavioral activation?

**General (After the intervention period)**

1. Were you able to continue and maintain the intervention bundle (BA and MO) on your own (after the intervention period)?
   1. Any barriers to continue these intervention bundle without the perioperative wellness partner?
   2. Any particular facilitator(s) that helped you to continue working in intervention bundle?
   3. Any suggestions to sustain the intervention bundle?
2. How confident do you feel that older adults having surgery at our hospital will accept and follow the intervention bundle?
3. What types of changes may we need to make for the intervention to be long-lived and available to all?
   1. Probe: What aspect of medication optimization and behavioral activation would make it difficult for you or someone else to adhere to it?
   2. Probe: How can we make sure that everyone independent of their socioeconomic status, race, ethnicity, gender, or other classification, is able to receive this intervention?

**Feasibility of Study Procedures**

1. What was your experience with the research procedures – at the time of screening/consent?
   1. Did you encounter any issues with the assessments when you started the study, 1-month, 2-month and 3-month follow-ups?
   2. Was there anything that you think did not work for you?
2. Did you ever think about withdrawing or not participating anymore in this study?
   1. If so, why? What were the reasons?
   2. If not, what made you continue to participate?
3. Any thoughts on the research and its impact?

Thank you for your generosity in time and effort!

**Semi-Structured Interview Guide: CAREGIVERS**

**Medication Optimization**

1. How effective was medication optimization for your care recipient <insert name>?
2. Do you think medication optimization helped them? If so, how
   1. Probe: Did it improve their mood?
   2. Probe: Did you and your care recipient have better control of their medications after medication optimization?
3. Prior to the intervention, did you and your care recipient know their full list of medications and why they were taking each one?
4. During medication optimization, were the changes to your care recipient’s medications discussed with you?
   1. What is your role in making sure the care recipient adhere to their medications?
5. Were there any barriers/side-effects in following the medication changes made by our perioperative wellness partner?
   1. If so, please describe these.
6. In your opinion, in your role as a caregiver, what were the advantages of medication optimization?
   1. How did it help with their anxiety and depression?
   2. What aspects of medication optimization were helpful for your care recipient?
   3. How did it impact you in your role?
      1. Caregiving is a hard job- did this add to your stress?
   4. In your opinion, what were the disadvantages of medication optimization?
   5. Do you have any suggestions on how to address these challenges?

**Behavioral Activation**

1. How effective was behavioral activation for your care recipient? Do you think it helped them?
   1. If so, how did it help?
   2. Did you see any changes to their mood since doing BA?
   3. What parts of BA helped – goal setting, value formulation, activity scheduling etc?
   4. What parts of BA that wasn’t helpful to the care recipient – and why?
   5. What is your role in making sure the care recipient comply to these activities?
2. Where did BA help your care recipient the most: pre-, post-operative periods?
   1. What did you like most about BA?
   2. Did they ever talk to you about their experiences with BA activities?
   3. Did they need your support to be motivated in BA?
3. Do you feel that the timing and frequency of sessions were appropriate for your care recipient?
   1. Did the sessions before surgery help them prepare for the surgery and ease your anxiety and stress around surgery?
   2. Did the sessions after surgery help their mental and physical recovery and well-being?
   3. Would you change the number of sessions or how often they had them (frequency)? Was it too little or too much at any point?
   4. How did it impact you in your role?
4. What were the disadvantages of behavioral activation?
   1. What made behavioral activation difficult to follow or adhere to by your care recipient?
   2. Did they feel overwhelmed with behavioral activation at any point?
   3. What are some suggestions you may have to improve the acceptability and feasibility of BA for others?
5. Do you have any other comments or suggestions regarding behavioral activation?

**S2. Fidelity rating checklist**

Participant ID

**Session info**

Date of session

Wellness Partner Conducting Session A

B


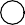

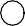

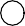


C

Fidelity Rating Method: a. Direct observation

b. Review of intervention session recording


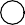

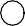

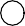


c. Review of intervention session documentation form

Fidelity Rated by:

Fidelity Rating date:

Session type: a. Medication Optimization only

b. Behavioral Activation only


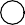

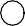

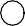


c. Medication Optimization and Behavioral Activation

BA session number: MO session number:

**Medication Optimization- Initial Assessment [MO Session #1]**

√ Topic Note details of all topics addressed

1. Complete detailed review of patient's home medications, verifying Epic entries with patient and/or caregiver. Record details in Medication Form.

2. Review medications against deprescribing list.

3. Review medications against dose-escalation list.

4. Discuss medication/s eligible for deprescribing. Get feedback for stopping. Find out who prescribes it and get buy-in to contact prescriber.

5. Discuss medication/s eligible for dose escalation Find out who prescribes it and get buy-in to contact prescriber.

**Medication Optimization- Initial Recommendation [MO Session #2]**

√ Topic Note details of all topics addressed Before session

1. Email medication list to the medication optimization contact along with details about patient experience, issues with medication adherence, resistance to stopping certain meds, perceived effectiveness, etc. Note any differences from their medication list in Epic.

2. Document the medication optimization recommendations or indicate that no changes were recommended (note who made them and date of correspondence).

3. Review medications against dose-escalation list.

4. How was the physician involved in the recommendation and decision-making? Record any responses received from the physician.

In session

5. Communicate recommendations to patient. Note date of communication and any questions or concerns.

**Medication Optimization- Follow-Up [MO Session #3-10]**

√ Topic Note details of all topics addressed

1. Review medication optimization recommendations.

2. Assess whether recommendations were implemented.

3. Note any improvements or problems. Respond positively and affirm the positive benefits of making this change

4. Assess for other medication changes since last session, including stopping a medication, taking a different dose, or taking a new medication?

After session

5. Update Medication Form

6. Update team of any changes

**Medication Optimization- 24hr Post-Operative [current surgical status = 24hr Post-Op]**

√ Topic Note details of all topics addressed

1. Check MAR in Epic appx 24 hours post-op to assess whether the patient's antidepressant treatment was held for surgery. If it was held, check to see if it has been re-started.

2. In the event that a held antidepressant is not re-started post-operatively, CPMH pharmacists will contact the patient's unit pharmacist. Record name of pharmacist, contact method and information, date of correspondence, details of correspondence.

**Behavioral Activation - Session 1 [BA Session #1]**

√ Topic

Note details of all topics addressed:

Opening

Review agenda with patient and seek patient's input. Agenda: Personalized Rationale; Tracking Everyday Activities

Brief symptom assessment of depression and anxiety

Personalized Rationale

1. Assess and discuss negative life experiences (including the upcoming surgery, and any other difficult circumstances).

2. For context and to build upon strengths, assess and discuss positive life experiences (what's been going well lately).

3. Assess and discuss emotional and behavioral responses (what are they doing, not doing, doing more of, doing less of in response to the negative life experiences? Are those responses causing any problems?)

4. Validate emotional and behavioral responses as natural, normal, common

5. Discuss symptom cycles and how behavioral responses can perpetuate problems. This is the idea that our natural behavioral responses to a problem or symptom can sometimes make the problem worse, or cause other problems.

6. Explain the goal of BA: activation as an alternative to the patient's behavioral responses. Changing what we do can change how we feel and think, even in difficult circumstances.

7. Seek feedback and verify understanding of the intervention rationale

8. Discuss interventionist role to coach, guide, and help think of strategies, acknowledging that there will be challenges.

9. Throughout - use the patient's language instead of jargon to discuss problems, responses, and symptoms. Make note of patient's preferred terms.

Tracking Everyday Activities (Activity Monitoring)

1. Introduce Tracking Everyday Activities form as an initial step in working together.

(Logging what you do each day can help to identify patterns and track progress on your goals. When we look for opportunities to interrupt your symptom cycle, we will be more effective if we have a shared understanding of your day-to-day life.)

2. Seek feedback and verify understanding.

3. Collaboratively set goal for # of days to track activities before the next session, aiming for a minimum of 2 days.

Concluding

1. Ask what questions or concerns the patient has.

2. Arrange date, time, and format for next session (appx 2 weeks, or sooner to allow for session 2 to occur before surgery).

3. Remind of goal to track activities.

4. Describe any other topics addressed during this session.

**Behavioral Activation - Session 2 [BA Session #2]**

√ Topic

Note details of all topics addressed:

Opening

1. Review agenda with patient and seek patient's input.

Agenda: Review & discuss "Tracking Everyday Activities". Complete "Choosing Your Wellness Goals." Schedule at least one goal.

2. Brief symptom assessment of depression and anxiety

Review Tracking Everyday Activities (Activity Monitoring)

1. Review in detail the days they tracked their activities. For phone sessions, record on your own version of the form. For in person, make a photocopy.

2. If they did not record their activities:

1. Explain the purpose of tracking activities.
2. Discuss and problem-solve barriers.
3. Review in detail their activities the previous day.

3. Assess how the patient's activities impacted their mood.

4. Ask whether the days tracked represent their typical daily routine.

5. Ask whether the days tracked represent their ideal daily routine.

Choosing Your Wellness Goals

1. Use the Choosing Your Wellness Goals form to identify potential goals. If the patient does not have the form, read each option to them.

2. Ask more about any goal they indicate is important to them. Make note of the details.

3. Prioritize 1 or 2 to work towards now.

Scheduling Your Wellness Goals.

1. Introduce the Scheduling form as a way to work towards the priorities they identified.

2. Collaboratively identify activities to work on

3. Consider difficulty of activity in the current context and break tasks into smaller parts if needed

4. Schedule at least one activity concretely (what, where, when, with whom)

5. Problem solve any obstacles to activity completion

6. Discuss using to continue to track activities; and to record progress towards the activity goal.

Concluding

1. Ask what questions or concerns the patient has.

2. Arrange date, time, and format for next session (appx 2 weeks, or sooner to allow for session 2 to occur

before surgery).

3. Remind of wellness goal and tracking.

4. Describe any other topics addressed during this session.

**Behavioral Activation - Sessions 3-9 [BA Session #3-9]**

√ Topic

Note details of all topics addressed:

Opening

1. Review agenda with patient and seek patient's input.

Agenda: Review & discuss wellness goals that were scheduled. Review & discuss activity tracking. Set new goals.

2. Brief symptom assessment of depression and anxiety

3. Check in on patient's health and recovery.

4. Assess emotional and behavioral responses.

5. Validate emotional and behavioral responses as natural, normal, common.

Review & Discuss Progress on Scheduled Wellness Goals

1. Review the goal/s the patient set at the previous session.

2. Ask whether they met their goal.

3. Commend any progress towards the goal.

4. Assess the impact of completing the activity. How did they feel during? After?

5. Identify and problem-solve any barriers to completing the scheduled activity.

Review & Discuss Activity Tracking

1. Review in detail the days they tracked their activities. For phone sessions, record on your own version of the form. For in person, make a photocopy.

2. If they did not record their activities:

- Explain the purpose of tracking activities.
- Discuss and problem solve barriers.
- Review in detail their activities the previous day.

3. Assess how the patient's activities impacted their mood.

4. Assess for routine disruptions (any disruptions affecting their normal routines and the reasons for disruptions).

5. Assess for avoidance behaviors (any behavior people use to escape or distract themselves from difficult thoughts, feelings, and situations).

6. Discuss using your Tracking Your Everyday Activities Form to continue to track activities and record progress towards the activity goal.

Schedule New Wellness Goals

Assess patient's primary concern and goals.

- 1. Collaboratively identify activities to work on (can revisit Choosing Your Wellness Goals form).
  2. Consider difficulty of activity in the current context and break tasks into smaller parts if needed
  3. Schedule at least one activity concretely (what, where, when, with whom). Use Scheduling Your Wellness Goals form or method of patient's choice.
  4. Problem solve any obstacles to activity completion

Concluding

1. Ask what questions or concerns the patient has.

2. Arrange date, time, and format for next session (appx 2 weeks, or sooner to allow for session 2 to occur

before surgery).

3. Remind of wellness goal and tracking.

4. Describe any other topics addressed during this session.

1. Introduce the Scheduling form as a way to work towards the priorities they identified.

2. Collaboratively identify activities to work on

3. Consider difficulty of activity in the current context and break tasks into smaller parts if needed

4. Schedule at least one activity concretely (what, where, when, with whom)

5. Problem solve any obstacles to activity completion

6. Discuss using to continue to track activities; and to record progress towards the activity goal.

Concluding

1. Ask what questions or concerns the patient has.

2. Arrange date, time, and format for next session (appx 2 weeks, or sooner to allow for session 2 to occur

before surgery).

3. Remind of wellness goal and tracking.

4. Describe any other topics addressed during this session.

√ Topic Note details of all topics addressed Opening

1. Review agenda with patient and seek patient's input.

Agenda: Review progress from initial session. Discuss what practices to continue. Assess need for ongoing mental health treatment and assist with referrals.

2. Brief symptom assessment of depression and anxiety

Termination Preparation

1. Review the goal/s the patient set at the previous session.

2. Review progress from initial session.

3. Assess and reinforce impact. Commend progress.

4. Discuss what practices the patient would like to 'keep,' and address any barriers to doing so.

5. Assess need for ongoing mental health treatment and assist with referrals.

6. Describe any other topics addressed during this session.

**Global Ratings [all sessions]**

**When possible, note concrete examples to support the rating, with approximate time stamp.**

1. Conveys warmth and empathy
2. Validates & normalizes.
3. Discussion is collaborative.
4. Discussion is concrete and focused on behavior.
5. Uses patient's language instead of jargon.
6. Encourages experimentation (try it and see).
7. Supportive
8. Responsive to patient's needs.

Never Rarely Sometimes Consistently Consistently &

Skillfully


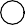

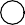

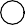

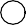

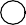


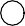

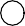

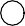

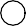

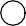

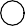

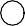

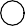

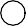

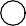

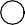

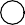

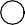

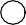

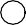


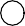

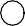

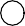

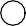

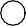


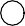

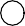

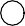

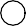

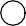


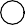

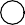

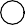

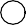

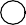

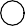

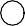

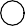

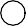

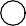


General Comments and feedback

Feedback: Please check all that apply

Feedback provided to wellness partner verbally

Feedback provided to wellness partner in writing

Session reviewed in detail at weekly intervention meeting

**S3. TIDieR checklist for reporting on intervention bundle.**

| **Item #** | **Item** | **Description** | **Location** | **Other details** |
| --- | --- | --- | --- | --- |
| 1 | Brief name | Provide a name or phrase that describes the intervention. | p. 21, line 22 | Surgical wellness program |
| 2 | Why | Describe any rationale, theory, or goal of the elements essential to the intervention. | p. 24, lines 4-9 |  |
| 3 | What | Materials: describe any physical or informational materials used in the intervention, including those provided to participants or used in intervention delivery or in training of intervention providers. Provide information on where the materials can be accessed. | Table 2; page 27, lines 2-3 | SOPs available upon request |
| 4 | Procedures | Describe each of the procedures, activities, and/or processes used in the intervention, including any enabling or support activities. | p.24, lines 1-19 |  |
| 5 | Who provided | For each category of intervention provider, describe their expertise, background, and any specific training given. | p.8, line 17; p. 18, lines 6-10 | Details on training also available in Table 2 |
| 6 | How | Describe the modes of delivery of the intervention and whether it was provided individually or in a group. | p. 24, lines 8-9, 20 |  |
| 7 | Where | Describe the type of location where the intervention occurred, including any necessary infrastructure or relevant features. | p. 24, lines 8-16 |  |
| 8 | When and how much | Describe the number of times the intervention was delivered and over what period of time including the number of sessions, their schedule, and their duration, intensity, or dose. | p. 24, lines 18-21 |  |
| 9 | Tailoring | If the intervention was planned to be personalized, titrated, or adapted, then describe what, why, when, and how. | p. 21-24, p. 25, lines 1-4 |  |
| 10 | Modifications | If the intervention was modified during the course of the study, describe the changes (what, why, when, and how). | N/A |  |
| 11 | How well: planned | If the intervention adherence or fidelity was assessed, describe how and by whom, and if any strategies were used to maintain or improve fidelity, describe them. | p. 11, lines 5-22; p. 12, lines 1-2 |  |
| 12 | How well: actual | If intervention adherence or fidelity was assessed, describe the extent to which the intervention was delivered as planned. | p. 19, lines 18-21; p. 20, lines 1-12 |  |
